# Preparation and In Vitro Evaluation of Aspartic/Alginic Acid Based Semi-Interpenetrating Network Hydrogels for Controlled Release of Ibuprofen

**DOI:** 10.3390/gels7020068

**Published:** 2021-06-09

**Authors:** Muhammad Suhail, Yi-Han Hsieh, Arshad Khan, Muhammad Usman Minhas, Pao-Chu Wu

**Affiliations:** 1 School of Pharmacy, Kaohsiung Medical University, 100 Shih-Chuan 1st Road, Kaohsiung City 80708, Taiwan; suhailpharmacist26@gmail.com; 2 Department of Biomedical Science and Environmental Biology, Kaohsiung Medical University, 100 Shih-Chuan 1st Road, Kaohsiung City 80708, Taiwan; irene93303@gmail.com; 3 Department of Pharmaceutics, Faculty of Pharmacy, Khawaja Fareed Campus (Railway Road), The Islamia University of Bahawalpur, Punjab 63100, Pakistan; arshadpharma77@gmail.com; 4 College of Pharmacy, University of Sargodha, Sargodha 40100, Pakistan; 5 Department of Medical Research, Kaohsiung Medical University Hospital, Kaohsiung City 80708, Taiwan; 6 Drug Development and Value Creation Research Center, Kaohsiung Medical University, Kaohsiung City 80708, Taiwan

**Keywords:** aspartic acid, alginic acid, acrylic acid, ethylene glycol dimethacrylate, hydrogel

## Abstract

Different combinations of polymers, aspartic acid (ASP), alginic acid (AL), and monomer acrylic acid (AA) were crosslinked in the presence of an initiator ammonium peroxodisulfate (APS) and cross-linker ethylene glycol dimethacrylate (EGDMA) to develop aspartic acid/alginic acid-co-poly(acrylic acid) (ASP/ALPAA) (semi-interpenetrating polymer network (SIPN)) hydrogels by the free radical polymerization technique for the controlled delivery of ibuprofen (IBP). Various studies such as dynamic swelling studies, drug loading, in vitro drug release and sol−gel analysis were carried out for the hydrogels. Higher swelling was observed at higher pH 7.4 as compared to lower pH 1.2, due to the presence of carboxylic groups of polymers and the monomer. Hence, pH-dependent swelling was exhibited by the developed hydrogels which led to a pH-dependent drug release and vice versa. The structural properties of the hydrogels were assessed by FTIR, PXRD, TGA, DSC, and SEM which confirmed the fabrication and stability of the developed structure. FTIR analysis revealed the reaction of both polymers with the monomer during the polymerization process and confirmed the overlapping of the monomer on the backbone of the both polymers. The disappearance of high intense crystalline peaks and the encapsulation of the drug by the hydrogel network was confirmed by PXRD. TGA and DSC showed that the developed hydrogels were thermally more stable than their basic ingredients. Similarly, the surface morphology of the hydrogels was analyzed by SEM and showed a smooth surface with few pores. Conclusively, ASP/ALPAA hydrogels have the potential to deliver IBP for a long period of time in a controlled way.

## 1. Introduction

Poly(aspartic acid) (ASP) is a synthetic polymer consisting of free carboxylic groups or amino groups based on natural amino acids [1]. It is water soluble, biodegradable, and non-toxic in nature; for these reasons, it has been considered a potential candidate for drug carriers [2]. Due to the presence of the carboxylic groups, poly(aspartic acid) exhibits the same response to pH and ionic strength as other ionic polymers and maximum swelling can be achieved by lowering the ionic strength or enhancing the pH of the medium. This behavior, known as the polyelectrolyte effect, stems from the ionization of carboxylic groups [3,4]. Ionization or deprotonation produces negative charges throughout the network, resulting in the confirmation of extended chains and globules to coil transition [5,6,7]. 

Alginic acids are natural polysaccharide polymers and their biological source is brown seaweed (Phaeophyceae). A dilute alkaline solution is extracted with the seaweed that solubilizes the alginic acid present. The treatment of the mineral acid results in a thick and viscous mass caused in the production of free alginic acid (AL) which can be converted to a salt of AL, known as sodium alginate, used in different pharmaceutical and biomedical fields. AL is a linear polymer consisting of D-mannuronic acid and L-guluronic acid residues which are arranged in block form in the polymer chain. Due to intermolecular binding, a high viscous “acid gel” is formed by the hydration of AL. The water molecules are entrapped physically inside the alginate matrix after gelation, but still can migrate freely. This all leads to great importance in many applications such as alginate gels for cell immobilization/encapsulation. The main characteristic of the holding of a high amount of water in the gel is due to capillary forces. Heat-stable gels can be developed at room temperature [8]. Acrylic acid (AA) is an aqueous, soluble and pH-sensitive polymer employed mostly in stimuli responsive polymeric carrier systems, especially in pH-sensitive hydrogels [9]. Due to its pH responsive nature, AA is used widely in different biomedical and pharmaceutical fields as it releases a high quantity of drug at the target site. Similar to other pH sensitive polymers, it exhibits a greater swelling index in basic medium as compared to acidic medium due to the deprotonation of functional groups (COOH), and hence, releases the drug in a greater quantity in basic medium [10,11] 

Hydrogels are three-dimensional structures possessing the ability to hold a greater amount of water or physiological medium due to the existence of a maximum number of hydrophilic groups of hydrophilic polymers [12,13]. Because of the strong physical or chemical linkages of the functional groups of the polymer chains, hydrogels absorb a greater amount of water without dissolving in it. Synthetic and natural polymers are used for the preparation of hydrogels via different crosslinking techniques [14,15]. Hydrogels in swelled form look similar to living tissues representing its rubbery and soft nature. Hydrogels protect the encapsulated drug from the unhealthy environment of body [13,16,17].

A semi-interpenetrating polymer network (SIPN) is the crosslinked network of two polymers where one polymer is crosslinked in the presence of another polymer, and persists there without producing any further noncovalent interaction between the two polymers [18]. SIPN hydrogels have the intermediary properties of both polymers physically and mechanically. When polymers with low water swellability and high mechanical strength are cross-linked with polymers of high swellability and low mechanical strength, this generates SIPN structures with intermediary features. The SIPN technique mostly helps to enhance the physical and mechanical properties of natural polymer-based hydrogels [19]. Currently, this method is used by a number of researchers to enhance the physical and mechanical strengths of biodegradable natural polymer-based hydrogels [20,21].

Ibuprofen (IBP), 2-(4-Isobutylphenyl) propionic acid is an NSAID that inhibits the cyclooxygenase system. Commonly, IBP is used as an analgesic and antipyretic for different inflammatory disorders [22]. It may be used as a short duration therapy, such as for a headache, or for chronic therapy, such as in cases of osteoarthritis and rheumatoid arthritis. The solubility of IBP is very low (~1 mg/mL) [21], and it is absorbed well (approximately 100%, with a high plasma concentration around 1–2 h after ingestion) when administered orally [23,24]. The half-life of IBP is 1.7–2 h and it is eliminated rapidly from the systemic circulation; hence, several dosages are required for an effective and long pharmacological action [25]. Additionally, the maintenance of analgesic effect is more important than the rapid onset of action when used for chronic disorders. Considering all this, different adverse effects such as gastric toxicity, including gastric irritation, bleeding, abdominal pain and ulcers are expected [26]. Therefore, to overcome the related adverse effects and achieve effective and prolonged pharmacological action of IBP, a polymeric system is required to release the IBP in a controlled way.

Here, we report on the development of ASP/ALPAA interpenetrating hydrogels by free radical polymerization technique for the controlled delivery of IBP. The fabricated hydrogels were assessed further for various studies such as dynamic swelling, drug loading, in vitro drug release and sol−gel analysis. Along with this, characterizations such as FTIR, PXRD, TGA and DSC, and SEM were carried out to uncover the structural arrangement, crystallinity, thermal stability and surface morphology of the ASP/ALPAA hydrogels. 

## 2. Results and Discussions

### 2.1. Dynamic Swelling

**Figure 1:**
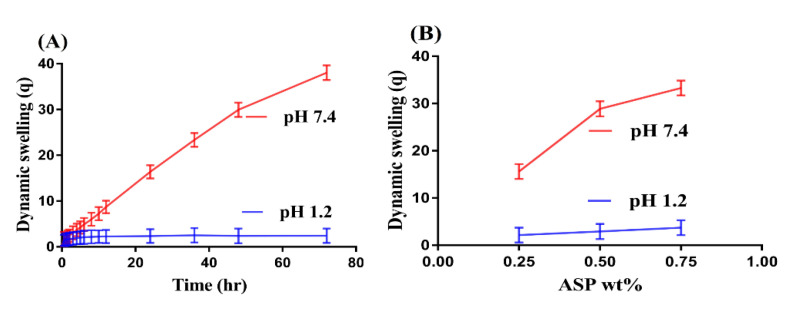

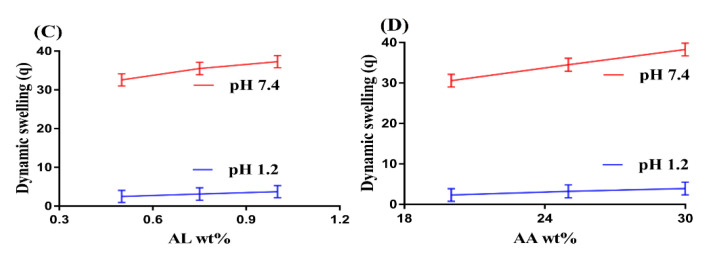
Effect of (**A**) pH, (**B**) Aspartic acid (ASP), (**C**) Alginate (AL), and (**D**) Acrylic acid (AA) on dynamic swelling of ASP/ALPAA hydrogels.

Swelling studies were conducted for ASP/ALPAA hydrogels at both acidic and basic media, i.e., at pH 1.2 and 7.4, respectively, as shown in Figure 1A–D. The pH value highly influences the dynamic swelling of developed hydrogels as maximum swelling is shown at a higher pH of 7.4 compared to a lower pH of 1.2 (Figure 1A). This behavior of the fabricated system is due to the deprotonation of functional groups of both polymers and monomers at higher pH. ASP contains COOH and NH^3+^ groups which are protonated at acidic pH 1.2. Similarly, AL and AA have COOH functional groups which are protonated at an acidic pH, i.e., pH 1.2. They form conjugate and strong hydrogen bonding, due to which the hydrogels network is collapsed. However, as the pH of the medium is increased, increase in deprotonation of COOH and NH^3+^ groups is observed, which leads to increase in charge density and thus generate strong electrostatic repulsive forces. This forces result in an increase in swelling of fabricated hydrogels and thus higher swelling is exhibited at higher pH [27,28].

Similarly, hydrogels contents also influence the swelling behavior of hydrogels at both pH 1.2 and 7.4 as shown in Figure 1B–D. Increase in swelling is observed with increase in the concentration of ASP (Figure 1B). The main reason is the increase in charge density due to the increase in COOH and NH^3+^ groups which leads to higher swelling of hydrogels [29]. As in ASP, a rise in swelling is observed when the concentration of AL (Figure 1C) is increased. AA has pKa value near to 4. Thus, as the concentration of AA is increased, COOH groups are generated in high concentration, which leads to enhance the hydrophilicity of the hydrogels network, and thus maximum swelling is observed (Figure 1D) and vice versa [30]. Conclusively, we can demonstrate that pH dependent swelling is shown by developed hydrogels network. 

### 2.2. Drug Loading Behavior

Drug loading is conducted for the purpose to determine the quantity of the drug loaded by the polymeric hydrogels as shown in Table 1. Drug loading mainly depends upon the swelling behavior of the hydrogels. If swelling is higher, maximum drug will be loaded by hydrogels and vice versa. Drug loading is enhanced as the concentration of ASP and AL is increased. Similarly, increase in drug loading is revealed by developed hydrogels as an increase in concentration of AA is detected. The maximum swelling of hydrogels depends on the porosity of the hydrogels, higher the porosity, greater will be the swelling and as a result higher will be drug loading [31] and vice versa. 

### 2.3. Dissolution Studies 

**Figure 2:**
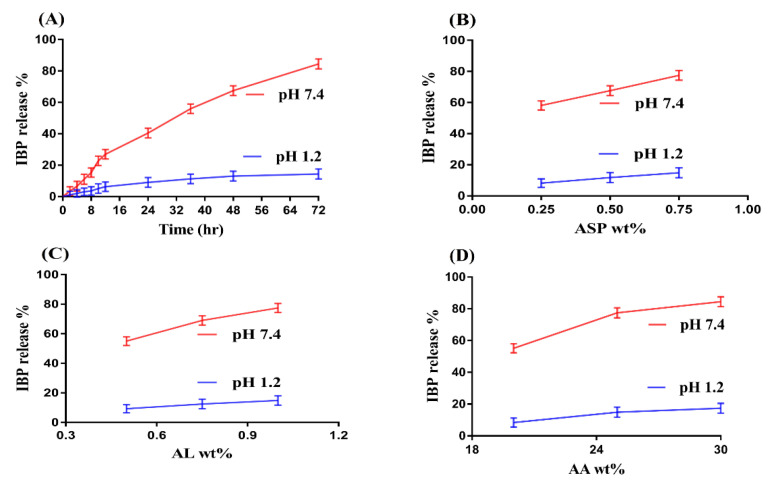
Effect of (**A**) pH, (**B**) Aspartic acid (ASP), (**C**) Alginate (AL), and (**D**) Acrylic acid (AA) on IBP release percent from ASP/ALPAA hydrogels.

Dissolution studies are performed to evaluate the percent release of drug at both pH 1.2 and 7.4 as shown in Figure 2A–D. Similar to swelling, pH also highly influences the percent drug release as greater percent drug release is observed at higher pH 7.4 compared to lower pH 1.2 (Figure 2A). The main reason is the hydrophilic functional groups of ASP, AL and AA. COOH groups of both polymers and monomer protonate in acidic medium, produce strong conjugate and hydrogen bonding, that leads to drop in swelling index and as a result percent drug release is reduced at pH 1.2. However, as the pH increases from 1.2 to 7.4, deprotonation of COOH functional groups of both polymers and monomer increases which produces strong electrostatic repulsive forces and leads to increase in swelling and thus percent drug release is increased [30,32,33] and vice versa.

The concentration of polymers and monomer also influences the percent drug release from ASP/ALPAA hydrogels at both pH 1.2 and 7.4 as shown in Figure 2B–D. Maximum percent drug release is observed at both pHs as the concentration of ASP and AL increases (Figure 2B,C). Similarly, increase in percent drug release is indicated as the concentration of AA (Figure 2D) increases and vice versa. This all mean that, similar to swelling, pH dependent percent drug release is observed by fabricated hydrogels due to the presence of COOH groups of ASP, AL and AA. Higher the swelling, greater will be the drug loading and maximum will be the percent drug release [30,34] and vice versa. 

### 2.4. Kinetic Modeling

Kinetic modeling was conducted to deduce the release mechanism of the drug from fabricated ASP/ALPAA hydrogels as indicated in Table 2. A suitable model was selected on the basis of “r” value close to 1. The “r” value is the regression coefficient. “r” values for zero order and first order are found within range of 0.9517–0.9936 and 0.8560–0.9908, respectively. Additionally, similarly, “r” values for Higuchi and Korsmeyer–Peppas models are found in the range of 0.9344–0.9960 and 0.9621–0.9964, respectively. It can be seen clearly from Table 2 that the “r” values of Korsmeyer–Peppas model are higher than other respective models, which demonstrate that all formulations of ASP/ALPAA hydrogels exhibit Korsmeyer–Peppas model. The type of diffusion process is confirmed by the release exponent “n” value. Fickian diffusion mechanism (*n* = 0.5) and non-Fickian or anomalous (*n* > 0.5). “*n*” values are observed in the range of 0.5132–0.7563 (Table 2) approving non-Fickian diffusion [35,36].

### 2.5. Sol−Gel Analysis

Sol−gel fraction is carried out for all formulations of ASP/ALPAA hydrogels as shown in Table 1. Sol is the uncross-linked soluble fraction while gel is the insoluble crosslinked fraction of the hydrogels. Gel fraction is increased with increase in the concentration of ASP and AL. Greater the concentration of polymers, higher will be the availability of free radicals for monomer, and faster will be the polymerization reaction. As the composition of ASP and AL increases, the polymerization reaction between ASP/ASP and AA increases, and thus gel fraction is increased [37] and vice versa. Similarly, increase in gel fraction is observed as the concentration of AA is enhanced. On other hand, there is an inverse proportion between gel and sol fraction. Increase in gel fraction leads to decrease in sol fraction. Thus, as the concentration of ASP, AL and AA increases, decrease in sol fraction is observed and vice versa. The key point is the fast and quick polymerization reaction among the hydrogels contents which results in an increase in gel fraction while decrease in sol fraction [38].

### 2.6. FTIR Analysis

**Figure 3:**
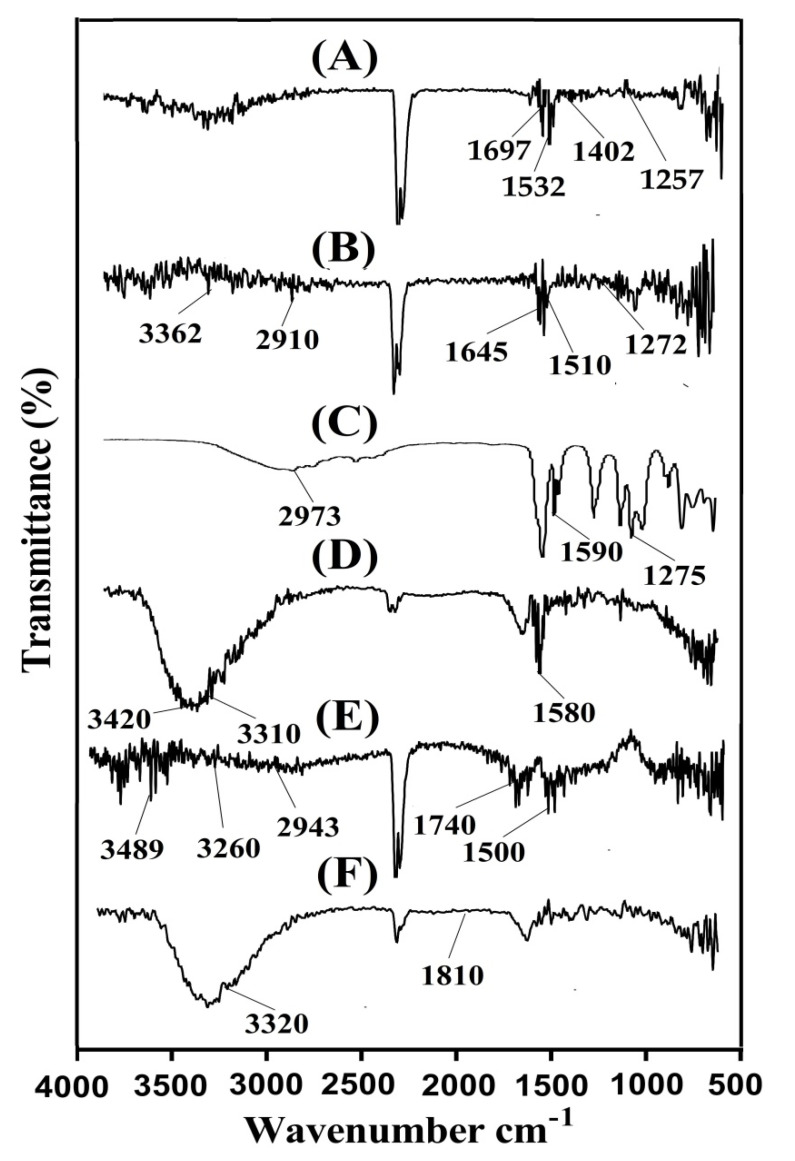
FTIR spectra of (**A**) Aspartic acid (ASP), (**B**) Alginate (AL), (**C**) Acrylic acid (AA), (**D**) unloaded ASP/ALPAA hydrogels, (**E**) Ibuprofen (IBP), and (**F**) drug-loaded ASP/ALPAA hydrogels.

FTIR is carried out to evaluate the structural arrangement of the constituents used in the preparation of a polymeric system. FTIR spectrum of ASP, AL, AA, unloaded ASP/ALPAA hydrogels, IBP, and drug-loaded ASP/ALPAA hydrogels is shown in Figure 3A–F. FTIR spectrum of ASP (Figure 3A) assigns symmetric stretching vibration of carboxylate group by a peak at 1402 cm^−1^. Amide-I, amide-II and amide-III reveals bands at 1697, 1532 and 1257 cm^−1^, respectively. Zhao et al. (2006) also reported the same spectra as like in our current studies which further support our observation [1]. AL FTIR spectra (Figure 3B) indicate symmetrical and asymmetrical vibration of carbonyl group (COO-) at 1510 and 1645 cm^−1^ while peaks at 1272 and 3362 cm^−1^ indicate stretching vibrations of OH group, respectively. The stretching vibration of aliphatic C-H group is assigned at peak 2910 cm^−1^, whereas peaks at 978 and 1042 cm^−1^ are assigned to the C-O-C and C-C-C of pyranic band, respectively [39]. Similarly, prominent bands of AA (Figure 3C) reveal stretching vibration of –CH_2_ and –C–C at 2973 and 1590 cm^−1^, while a broad peak at 1275 cm^−1^ indicates stretching vibration of –C=O, respectively [40]. A change in position of different functional groups of ASP, AL and AA is seen in FTIR spectra of unloaded ASP/ALPAA hydrogels (Figure 3D) due to electrostatic interaction between them. Prominent peaks of ASP and AL at 1402, 1532 cm^−1^ and 1645, 3362 cm^−1^ are modified to 1450, 1580, 1708, and 3420 cm^−1^ peaks of unloaded ASP/ALPAA hydrogels. Similarly, prominent peaks of AA at 1275, 1590 and 2973 cm^−1^ are changed to 1330, 1634, and 3310 cm^−1^, respectively. Few peaks are disappeared while some new peaks are formed. The disappearance, formation and modification of peaks reveal the change in the intensity of the ASP, AL and AA peaks. This all indicate the fabrication of ASP/ALPAA hydrogels due to the overlapping of AA on the backbone of the ASP and AL. FTIR spectra of IBP (Figure 3E) assigns a prominent peak round at 1740–1835 cm^−1^ which represents the carbonyl stretching of isopropanoic acid groups. Characteristic peaks at 2943, 3260 and near to 3489 cm^−1^ correspond to carboxylic acid, OH stretching and aromatic stretching of CH groups. A peak at 1500 cm^−1^ assigns to C–C stretching [41,42]. The FTIR spectrum of drug-loaded ASP/ALPAA hydrogels (Figure 3F) shows a slight change in the position of the prominent peaks of drug due to loading of drug by the fabricated hydrogels. The prominent bands of the drug at 1740 and 3260 cm^−1^ are slightly changed to 1810 and 3320 cm^−1^ in loaded ASP/ALPAA hydrogels. The discussion demonstrates that drug is loaded by ASP/LAPAA hydrogels without any kind of interaction between them.

### 2.7. PXRD Study

**Figure 4:**
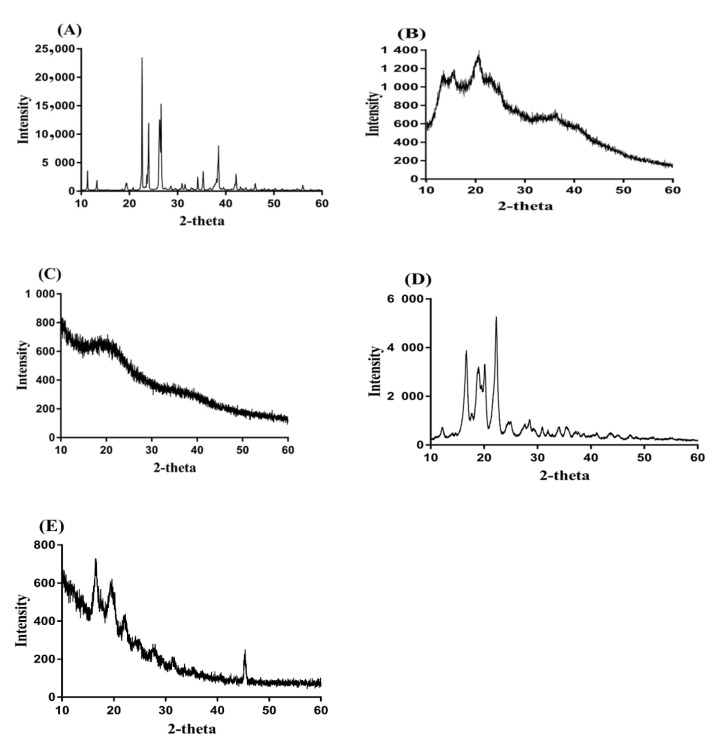
PXRD of (**A**) Aspartic acid (ASP), (**B**) Alginate (AL), (**C**) unloaded ASP/ALPAA hydrogels, (**D**) Ibuprofen (IBP), and (**E**) drug-loaded ASP/ALPAA hydrogels.

PXRD is performed for ASP, AL, unloaded ASP/ALPAA hydrogels, IBP, and drug-loaded hydrogels as shown in Figure 4A–E. PXRD of ASP (Figure 4A) indicates characteristic crystalline peaks of high intensity at 2θ = 22.04°, 25.65°, 27.30°, and 39.50° whereas prominent peaks of AL (Figure 4B) indicate PXRD at 2θ = 12.09°, 14.53°, 21.08°, and 37.51°, respectively. PXRD of unloaded ASP/ALPAA hydrogels is indicated in Figure 4C, where we can see that the intensity of crystalline characteristic peaks of ASP and AL is reduced due to the polymerization reaction among the hydrogel contents. Similarly, the characteristic crystalline peaks of the IBP (Figure 4D) are assigned at 2θ = 16.50°, 18.10°, 19.99°, and 21.70°, respectively. The intensity of broad crystalline peaks of the drug is reduced by ASP/ALPAA hydrogels (Figure 4E) [43]. The PXRD of the unloaded and drug-loaded ASP/ALPAA hydrogels is very close to each other and the slight difference is because of the loading of the drug by the ASP/ALPAA hydrogels [44].

### 2.8. TGA Analysis

**Figure 5:**
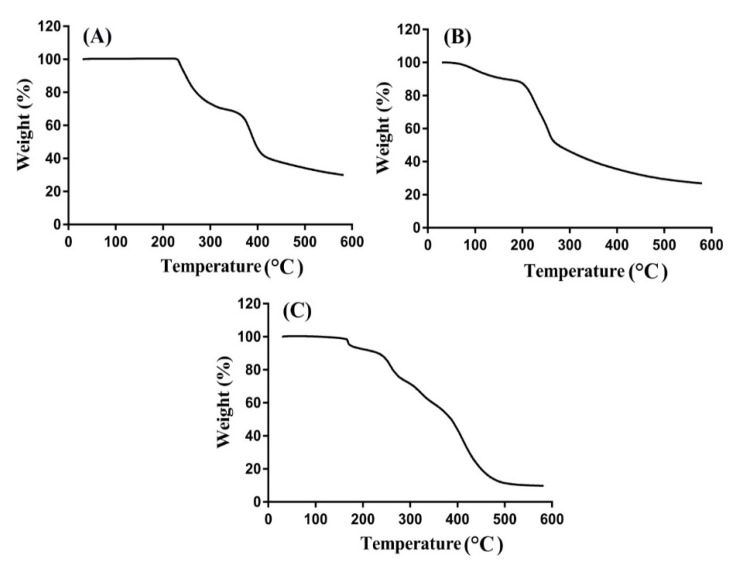
TGA of (**A**) Aspartic acid (ASP), (**B**) Alginate (AL), and (**C**) ASP/ALPAA hydrogels.

TGA was carried out to analyze the thermal stability of the unreacted hydrogels contents and developed hydrogels individually. Therefore, TGA is carried out for ASP, AL and ASP/ALPAA hydrogels as shown in Figure 5A–C. TGA of ASP (Figure 5A) indicates weight reduction of 25% within temperature range of 205–300 °C. As temperature approaches to 380 °C, further decrease of 15% is perceived in weight of ASP. After that a rapid decline is seen in weight reduction in ASP and degradation of ASP starts at 400 °C due to degradation of amino and carboxyl functional groups of ASP molecules [45]. Weight reduction in AL is shown at three stages by TGA as shown in Figure 5B. Initially 17% weight is reduced as temperature approaches to 185 °C. After that, 34% weight reduction is observed within range of 188–248 °C due to loss of moisture. Further reduction of 12% is detected in the weight of AL till temperature reaches to 405 °C and degradation of AL is started then [46]. A 20% decrease in weight of ASP/ALPAA hydrogels (Figure 5C) is seen within temperature range of 70–165 °C. Similarly, as the temperature increases, further decrease in weight of developed hydrogels is perceived as 21% weight reduction is observed at temperature 260 °C. Finally, 40% weight is lost as temperature approaches to 500 °C and degradation of ASP/ALPAA hydrogels is started onward. The discussion indicates that thermal stability of ASP/ALPAA hydrogels is higher than unreacted pure ASP and AL. The degradation half-life of ASP/ALPAA hydrogels (t_1/2_ = 500 °C) which is higher than degradation half-lives of ASP and AL, i.e., ASP (t_1/2_ = 400 °C) and AL (t_1/2_ = 405 °C), respectively. The greater thermal stability of the developed hydrogel reveals the strong cross-linking and grafting reaction among polymers, monomer and cross-linker. Barket et al. (2018) prepared PEG 4000- based hydrogels and found greater thermal stability for developed network compared to unreacted polymer [47]. 

### 2.9. DSC Analysis

**Figure 6:**
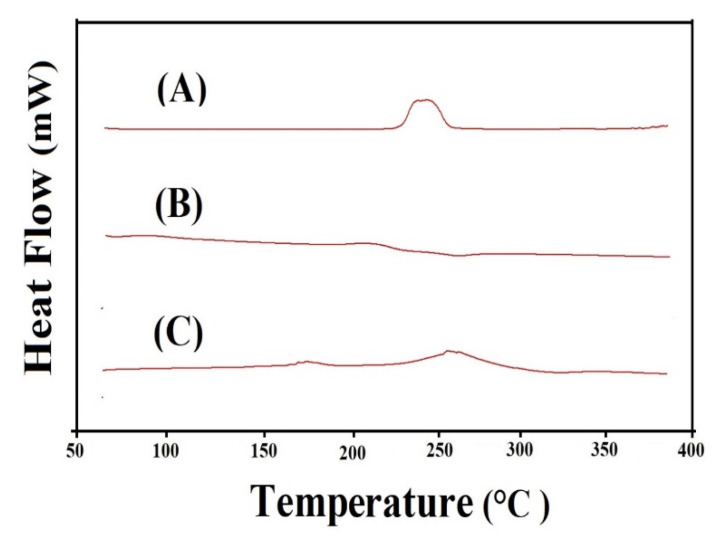
DSC of (**A**) Aspartic acid (ASP), (**B**) Alginate (AL), and (**C**) ASP/ALPAA hydrogels.

DSC is conducted for ASP, AL, and ASP/ALPAA hydrogels as shown in Figure 6A–C, respectively. DSC of ASP (Figure 6A) indicates an exothermic peak at 250 °C while an endothermic peak is observed at 248 °C concerned with loss of moisture. The exothermic peak at 250 °C indicates the degradation of ASP [48]. Similarly, two endothermic peaks at 63 and 268 °C are assigned by DSC of AL (Figure 6B) while an exothermic peak is detected at 237 °C, respectively. The endothermic peaks are concerned with water loss related to hydrophilic groups whereas exothermic peak shows the dehydration and depolymerization reactions [49]. DSC thermogram of ASP/ALPAA hydrogels (Figure 6C) reveals endothermic and exothermic peaks at 225, 330 and 170, 252 °C, respectively. The endothermic peak of ASP and AL at 248 and 268 °C is modified to 225 and 330 °C while the peaks at 250 and 237 °C indicating the exothermic peak of ASP and AL are shifted to 170 and 330 °C, respectively, in APS/ALPAA hydrogels. The above discussion indicates that fabricated hydrogels have higher thermal stability than unreacted ASP and AL and could be employed for controlled drug delivery system. Barkat et al. (2017) prepared chondroitin sulfate-based hydrogels and reported maximum thermal stability for the prepared network of hydrogels compared to hydrogels contents [50], which further supports our observation.

### 2.10. SEM Study

**Figure 7:**
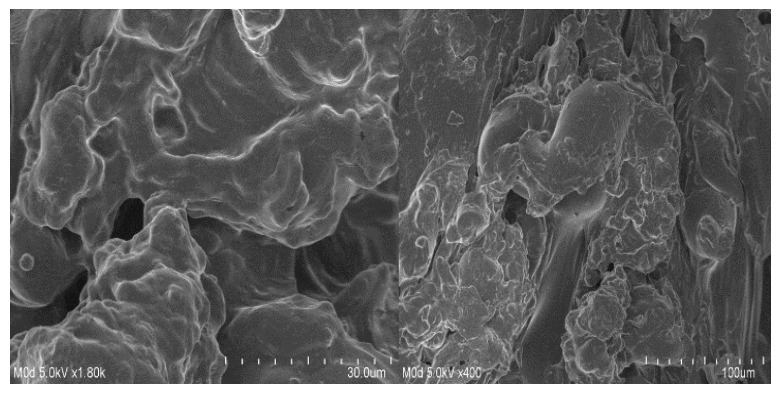
Surface morphology of ASP/ALPAA hydrogels.

SEM is carried out to evaluate and examine the surface morphology of the ASP/ALPAA hydrogels. A smooth surface with few pores is seen at various magnifications as shown in Figure 7. The pores provide channels for the water molecules, and as a result swelling, drug loading and drug release from the developed hydrogels is perceived [51,52]. 

### 2.11. Porosity Studies

**Figure 8:**
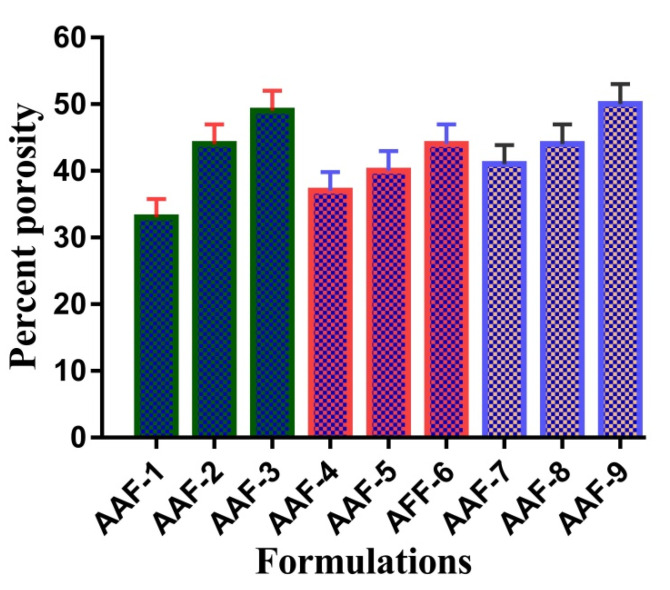
Percent porosity of ASP/ALPAA hydrogels.

Porosity of a system depends on the number of pores present in its structure. Percent porosity for all formulations of ASP/ALPAA hydrogels is shown in Figure 8. It is demonstrated that as the concentration of ASP and AL increases, increase in porosity of the hydrogels is observed. Viscosity of polymers affects the porosity of the developed hydrogels. As the viscosity of the system is increased, the removal of bubbles from the polymeric hydrogels is decreased up to certain extent, which generate pores in the fabricated hydrogels and as a result porosity is increased [11]. Similarly, porosity of hydrogels is increased as the concentration of AA is increased and vice versa.

## 3. Conclusions

The novelty of the current study is the development of ASP/ALPAA hydrogels by free radical polymerization technique for controlled delivery of IBP which could have various applications that are not limited to pharmaceutical drug delivery systems. Moreover, drug loading and release studies are being reported first time by this research work. The developed hydrogels exhibited maximum dynamic swelling and percent drug release at pH 7.4 compared to pH 1.2 due to the presence of COOH groups of ASP, AL, and AA which deprotonated as the pH of the medium enhances. FTIR analysis confirmed the structural arrangement of the ingredients used in the preparation of hydrogels. The crystallinity of the drug was reduced and encapsulated by fabricated hydrogels as indicated by PXRD. TGA and DSC revealed the greater thermal stability of the hydrogels as compared to basic ingredients. Similarly, SEM indicated a smooth surface with few pores of hydrogels. Thus, keep in view the results, the ASP/ALPAA hydrogels could be used as a potential candidate for controlled drug delivery system. 

## 4. Material and Methods

### 4.1. Materials 

Ibuprofen (IBP), alginic acid (AL) (Purity = 19 to 25% (carboxyl groups), MW = 10,000–600,000), aspartic acid (ASP) (Purity = 99 plus%, MW = 133.10) and acrylic acid (AA) (Purity = 98% extra pure, MW = 72.06 g/mol) were obtained from Across Organic (Carlsbad, CA, USA). Ammonium peroxodisulfate (APS) (Purity = 98%, MW = 228.21) was purchased from Showa (Tokyo, Japan). Similarly, ethylene glycol dimethacrylate (EGDMA) (Purity = 98%, MW = 198.22 g/mol) was purchased from Alfa-Aesar (Tewksbury, MA, USA).

### 4.2. Fabrication of ASP/ALPAA Hydrogels

Different concentrations of polymers ASP, AL and monomer AA were cross-linked in the presence of initiator APS and cross-linker EGDMA by free radical polymerization technique for development of aspartic/alginic acid-co-poly(acrylic acid) (ASP/ALPAA) hydrogels as shown in Table 3. A precise quantity of both polymers ASP and AL was taken and dissolved in deionized distilled water. Similarly, a weighed amount of AA, APS and EGDMA was taken. APS was dissolved in distilled water while AA and EGDMA were already present in liquid form. Initially, APS solution was added to AL solution, stirred for 5 min, and then the solution of AL and APS was added dropwise into the ASP solution, followed by AA addition. The mixture was kept on stirring for 20 min at room temperature. Finally, EGDMA was poured into mixture, kept it on stirring until a transparent solution was formed. The transparent solution was subjected to nitrogen gas in order to remove any dissolved oxygen in the solution. After that, the transparent solution was transferred into the glass tubes and placed in water bath at 60 °C for initial 2 h, and then enhanced the temperature up to 65 °C for next 21 h. The prepared gel was cut into 8 mm discs. Mixture of water and ethanol (50:50 *v/v*) was used for washing of gels disc to remove any unreacted content attached with the surface of gels. The developed discs of gel were placed at room temperature for 24 h initially and then placed in vacuum oven at 40 °C for one week. The prepared dried gels were subjected for further studies. 

### 4.3. Dynamic Swelling 

Dynamic swelling studies were carried out to evaluate the swelling behavior and pH-sensitivity of hydrogels. Hence, a weighed amount of the hydrogel discs was dipped in both acidic and basic media, i.e., pH 1.2 and 7.4 at 37 °C, respectively. Then, discs were taken out from the respective media after a regular interval of time, blotted with filter paper to remove excess of medium and weighed again. This process was continued until an equilibrium weight was obtained. This study was carried out triplicate [53]. Dynamic swelling was calculated by:

(1) $$\left(q\right)=\frac{{\;T}_{2}}{{\;T}_{1}}$$

where q = dynamic swelling, T_1_ = initial weight of hydrogels disc before swelling, and T_2_ = final weight of hydrogels disc after swelling at time t.

### 4.4. Drug Loading Behavior

Absorption and diffusion method were used for loading of IBP by developed hydrogels. Hydrogels discs of 8 mm of all formulations were immersed separately in 100 mL drug solution of 1% in phosphate buffer pH 7.4 for 72 h. Phosphate buffer pH 7.4 was selected as solvent for drug loading is because of high solubility of drug and maximum swelling of hydrogel in this solvent which leads to higher swelling and loading of drug by developed hydrogels. After 72 h, the discs of hydrogel were taken out and washed by distilled water to remove the surface entrapped drug from fabricated hydrogels. Then, the discs were placed in vacuum oven at 40 °C for dehydrating [54]. 

Estimation of drug loaded by hydrogels was performed by weight method. In this method, the weight of dried discs of hydrogels was measured before and after immersing in drug solution. Then, the weight of dried discs of unloaded hydrogels was subtracted from the weight of dried discs of loaded hydrogels [55] as shown in the following equation:

Amount of Drug loaded = Q_LD_ − Q_ULD_ (2)

where Q_LD_ = weight of dried loaded hydrogels disc, and Q_ULD_ = weight of dried unloaded hydrogels disc.

### 4.5. Dissolution Studies 

Dissolution studies were conducted for all formulations of ASP/ALPAA hydrogels to evaluate the pH-dependent percent release of drug at both acidic and basic media, i.e., pH 1.2 and 7.4, respectively. Loaded discs of hydrogel (height; 8 mm, and diameter; 10 mm) were immersed in 900 mL buffer solution of pH 1.2 and 7.4 using USP dissolution apparatus II (Sr8plus Dissolution Test Station) at 37 ± 0.5 °C. A 5 ml sample was taken after regular interval of time, and same amount of fresh medium was added back to maintain the sink condition constant. The collected samples were then analyzed by using UV- at a wavelength (λ_max_) of 222 nm in triplicate [56].

### 4.6. Kinetic Modeling

Various kinetic models such as zero-order, first-order, Higuchi and Korsmeyer–Peppas models were computed by evaluating release data of all formulation of ASP/ALPAA hydrogels [57]. 

### 4.7. Sol−Gel Analysis

Sol−gel analysis was performed for developed hydrogels to analyze the soluble uncross-linked and insoluble crosslinked part of hydrogel. The soluble uncross-linked part is known as sol fraction while the insoluble crosslinked part is known as gel fraction. Soxhelt extraction process was carried out for sol−gel analysis. The weighed amount of hydrogels disc was placed in a round bottom flask containing a specific quantity of deionized distilled water. A condenser was connected to round bottom flask. The Soxhelt extraction process was carried at 85 °C for 12 h. After that, the extracted hydrogels disc was placed in vacuum oven at 40 °C till complete dehydration. The dried hydrogels disc weighed again [58]. Sol−gel fraction was computed by the following equations:

(3) $$\mathrm{Sol}\;\mathrm{fraction}\;\%=\frac{{\;R}_{1}-{\;R}_{2}}{R_{2}}\times 100$$

(4) Gel fraction=100−Sol fraction

R_1_ shows the initial weight of hydrogels (prior extraction), and R_2_ represents the final weight of dried hydrogels (latter extraction).

### 4.8. Fourier Transform Infrared Spectroscopy (FTIR)

FTIR was carried out to confirm the fabrication of ASP/ALPAA hydrogels. FTIR was conducted for ASP, AL, AA, unloaded ASP/ALPAA hydrogels, IBP, and drug-loaded ASP/ALPAA hydrogels, respectively. Therefore, ATR (Attenuated total reflectance) method was used for spectra analysis. The samples were milled properly up to desired particle size and then Nicolet 380 FTIR (Thermo Fisher Scientific, Ishioka, Japan) was used for analysis and evaluation of samples. Number of scans and resolution were kept 8 and 4 cm^−1^ throughout the study, respectively. FTIR spectra were kept in the range of 4000–500 cm^−1^ [59].

### 4.9. Powder X-ray Diffractometry (PXRD) Study

PXRD pattern of IBP, unloaded ASP/ALPAA hydrogels and drug-loaded ASP/ALPAA hydrogels was analyzed by XRD-6000 Shimadz, Tokyo, Japan at room temperature. Drug and hydrogel discs were hold by plastic sample holder while the surface level of all samples was smoothened by a glass slide. For sample analyses, the theta (θ) was kept between 10°–60° at a rate of 2° 2θ/min at room temperature [60].

### 4.10. Thermogravimetric Analysis (TGA)

TGA was carried out for ASP, AL, and ASP/ALPAA hydrogels by using PerkinElmer Simultaneous Thermal Analyzer STA 8000. The desired particle size of hydrogels samples were obtained by passing the crushed samples through 40 meshes. For TGA thermogram, 0.5–5 mg sample was placed in an open pan connected to a microbalance. All the samples were heated from 40–600 °C below dry nitrogen [61]. 

### 4.11. Differential Scanning Calorimetry (DSC)

DSC was carried out for ASP, AL, and ASP/ALPAA hydrogels while using PerkinElmer DSC 4000 (Waltham, MA, USA). The specific amount of 0.5–3 mg of all samples was placed in an aluminum pan. Temperature, heating rate and purging of nitrogen was kept 50–400 °C, 20 °C/min, and 20 mL/min constant, respectively, during the whole study [62]. 

### 4.12. Scanning Electron Microscopy (SEM)

Surface morphology of ASP/ALPAA hydrogels was analyzed by SEM (JSM-5300 model, Jeol Ltd., Tokyo, Japan). Scanning of developed hydrogels was carried out at different magnifications [62].

### 4.13. Porosity Studies

Porosity studies were carried out by solvent replacement method to evaluate and analyze the porosity of hydrogel discs. Weighed dried (R_1_) discs of all formulations of ASP/ALPAA hydrogels were immersed in absolute ethanol of purity >99.9% for 48 h. After 48 h, discs of hydrogels were removed, blotted with tissue paper to remove excess solvent and accurately weighed (R_2_). Thickness and diameter of discs were also measured. Hydrogel porosity was calculated by using the following formula:

(5) $$\mathrm{Porosity}\;\mathrm{percentage}\;(\%)=\;\;\frac{R_{2}-R_{1}}{\rho V}\times 100$$

*ρ* refers to the density of absolute ethanol, and V is the volume of hydrogel after swelling. 

## Figures and Tables

**Table 1:** Sol−gel analysis and drug loading of ASP/ALPAA hydrogels.

Formulation Code	Sol Fraction %	Gel Fraction %	Drug-Loaded (mg)/350 mg of Dry Gel Weight Method
AAF-1	12.75	87.25	163.12 ± 1.10
AAF-2	09.71	90.29	184.42 ± 1.17
AAF-3	07.84	92.16	196.84 ± 0.97
AAF-4	13.10	86.90	166.18 ± 1.22
AAF-5	11.60	88.40	176.19 ± 1.03
AAF-6	09.71	90.29	184.42 ± 1.17
AAF-7	12.23	87.77	167.93 ± 1.01
AAF-8	09.71	90.29	184.42 ± 1.17
AAF-9	06.98	93.02	208.31 ± 1.13

**Table 2:** Kinetic modeling release of drug from ASP/ALPAA hydrogels.

F. Code	Zero Order r^2^	First Order r^2^	Higuchi r^2^	Korsmeyer–Peppas	
				r^2^	n
AAF-1	0.9533	0.9775	0.9344	0.9621	0.5242
AAF-2	0.9920	0.9840	0.9538	0.9950	0.6342
AAF-3	0.9832	0.9773	0.9826	0.9838	0.5132
AAF-4	0.9863	0.9771	0.9447	0.9866	0.5372
AAF-5	0.9903	0.9908	0.9780	0.9964	0.6134
AAF-6	0.9920	0.9840	0.9538	0.9950	0.6342
AAF-7	0.9517	0.8560	0.9960	0.9699	0.7563
AAF-8	0.9920	0.9840	0.9538	0.9950	0.6342
AAF-9	0.9936	0.9521	0.9754	0.9954	0.7413

**Table 3:** Feed ratio scheme for formulation of APS/ALPAA hydrogels.

F. Code	Polymer (Aspartic Acid) g/100 g	Polymer (Alginic Acid) g/100 g	Monomer (Acrylic Acid) g/100 g	Initiator (APS) g/100 g	Cross-Linker (EGDMA) g/100 g
AAF-1	0.25	1.00	25	0.5	0.5
AAF-2	0.50	1.00	25	0.5	0.5
AAF-3	0.75	1.00	25	0.5	0.5
AAF-4	0.50	0.50	25	0.5	0.5
AAF-5	0.50	0.75	25	0.5	0.5
AAF-6	0.50	1.00	25	0.5	0.5
AAF-7	0.50	1.00	20	0.5	0.5
AAF-8	0.50	1.00	25	0.5	0.5
AAF-9	0.50	1.00	30	0.5	0.5

## Data Availability

Did not report any data.

## References

[1] Zhao Y., Kang J., Tan T.W. (2006). Salt-, pH- and temperature-responsive semi-interpenetrating polymer network hydrogel based on poly(aspartic acid) and poly(acrylic acid). Polymer.

[2] Ravichandran R., Venugopal J.R., Sundarrajan S., Mukherjee S., Sridhar R., Ramakrishna S. (2012). Composite poly-L-lactic acid/poly-(alpha, beta)-DL-aspartic acid/collagen nanofibrous scaffolds for dermal tissue regeneration. Mater. Sci. Eng. C Mater. Biol. Appl..

[3] Zhao Y., Su H.J., Fang L., Tan T.W. (2005). Superabsorbent hydrogels from poly(aspartic acid) with salt-, temperature- and pH-responsiveness properties. Polymer.

[4] Sharma S., Dua A., Malik A. (2014). Polyaspartic acid based superabsorbent polymers. Eur. Polym. J..

[5] Yang J., Fang L., Tan T.W. (2006). Synthesis and characterization of superabsorbent hydrogels composites based on polysuccinimide. J. Appl. Polym. Sci..

[6] Senol S., Akyol E. (2020). Preparation and characterization of pH-sensitive hydrogels from photo-crosslinked poly (ethylene glycol) diacrylate incorporating titanium dioxide. Mater. Sci. Pol..

[7] Ali L., Ahmad M., Aamir M.N., Minhas M.U., Shah H.H., Shah M.A. (2020). Cross-linked pH-sensitive pectin and acrylic acid based hydrogels for controlled delivery of metformin. Pak. J. Pharm. Sci..

[8] Tonnesen H.H., Karlsen J. (2002). Alginate in drug delivery systems. Drug Dev. Ind. Pharm..

[9] Al-Tabakha M.M., Khan S.A., Ashames A., Ullah H., Ullah K., Murtaza G., Hassan N. (2021). Synthesis, Characterization and Safety Evaluation of Sericin-Based Hydrogels for Controlled Delivery of Acyclovir. Pharmaceuticals.

[10] Suhail M., Fang C.W., Minhas M.U., Wu P.C. (2021). Preparation, Characterization, Swelling Potential and In-Vitro Evaluation of Sodium Poly (Styrene Sulfonate)-Based Hydrogels for Controlled Delivery of Ketorolac Tromethamine. Pharmaceuticals.

[11] Zia M.A., Sohail M., Minhas M.U., Sarfraz R.M., Khan S., de Matas M., Hussain Z., Abbasi M., Shah S.A., Kousar M. (2020). HEMA based pH-sensitive semi IPN microgels for oral delivery; a rationale approach for ketoprofen. Drug Dev. Ind. Pharm..

[12] Hoare T.R., Kohane D.S. (2008). Hydrogels in drug delivery: Progress and challenges. Polymer.

[13] Hennink W.E., van Nostrum C.F. (2012). Novel crosslinking methods to design hydrogels. Adv. Drug Deliv. Rev..

[14] Buwalda S.J., Boere K.W.M., Dijkstra P.J., Feijen J., Vermonden T., Hennink W.E. (2014). Hydrogels in a historical perspective: From simple networks to smart materials. J. Control. Release.

[15] Ratner B.D., Hoffman A.S. (1976). Synthetic hydrogels for biomedical applications. Hydrogels for Medical and Related Applications.

[16] Hamidi M., Azadi A., Rafiei P. (2008). Hydrogel nanoparticles in drug delivery. Adv. Drug Deliv. Rev..

[17] Suhail M., Rosenholm J.M., Minhas M.U., Badshah S.F., Naeem A., Khan K.U., Fahad M. (2019). Nanogels as drug-delivery systems: A comprehensive overview. Ther. Deliv..

[18] Bhattarai N., Gunn J., Zhang M. (2010). Chitosan-based hydrogels for controlled, localized drug delivery. Adv. Drug Deliv. Rev..

[19] Sperling L.H. (2012). Interpenetrating Polymer Networks and Related Materials.

[20] Rao K.S.V.K., Naidu B.V.K., Subha M.C.S., Sairam M., Aminabhavi T.M. (2006). Novel chitosan-based pH-sensitive interpenetrating network microgels for the controlled release of cefadroxil. Carbohyd. Polym..

[21] Kulkarni R.V., Sreedhar V., Mutalik S., Setty C.M., Sa B. (2010). Interpenetrating network hydrogel membranes of sodium alginate and poly(vinyl alcohol) for controlled release of prazosin hydrochloride through skin. Int. J. Biol. Macromol..

[22] Sweetman S.C. (2009). Martindale: The Complete Drug Reference.

[23] Zhu K.J., Li Y., Jiang H.L., Yasuda H., Ichimaru A., Yamamoto K., Lecomte P., Jerome R. (2005). Preparation, characterization and in vitro release properties of ibuprofen-loaded microspheres based on polylactide, poly(epsilon-caprolactone) and their copolymers. J. Microencapsul..

[24] Kumar S.S., Rajkumar S., Ruckmani K. (2003). Formulation and evaluation of ibuprofen loaded nanoparticles for improved anti-inflammatory activity. ACTA Pharm. Sci..

[25] Potthast H., Dressman J.B., Junginger H.E., Midha K.K., Oeser H., Shah V.P., Vogelpoel H., Barends D.M. (2005). Biowaiver monographs for immediate release solid oral dosage forms: Ibuprofen. J. Pharm. Sci..

[26] Moore N. (2007). Ibuprofen: A journey from prescription to over-the-counter use. J. Roy. Soc. Med..

[27] Sohail M., Ahmad M., Minhas M.U., Ali L., Munir A., Khalid I. (2014). Synthesis and Characterization of Graft PVA Composites for Controlled Delivery of Valsartan. Lat. Am. J. Pharm..

[28] Lim S.L., Tang W.N.H., Ooi C.W., Chan E.S., Tey B.T. (2016). Rapid swelling and deswelling of semi-interpenetrating network poly(acrylic acid)/poly(aspartic acid) hydrogels prepared by freezing polymerization. J. Appl. Polym. Sci..

[29] Chen S.C., Wu Y.C., Mi F.L., Lin Y.H., Yu L.C., Sung H.W. (2004). A novel pH-sensitive hydrogel composed of N,O-carboxymethyl chitosan and alginate cross-linked by genipin for protein drug delivery. J. Control. Release.

[30] Sanli O., Ay N., Isiklan N. (2007). Release characteristics of diclofenac sodium from poly(vinyl alcohol)/sodium alginate and poly(vinyl alcohol)-grafted-poly(acrylamide)/sodium alginate blend beads. Eur. J. Pharm. Biopharm..

[31] Murthy P.S.K., Mohan Y.M., Sreeramulu J., Raju K.M. (2006). Semi-IPNs of starch and poly(acrylamide-co-sodium methacrylate): Preparation, swelling and diffusion characteristics evaluation. React. Funct. Polym..

[32] Kulkarni R.V., Sa B. (2009). Polyacrylamide-Grafted-Alginate-Based pH-Sensitive Hydrogel Beads for Delivery of Ketoprofen to the Intestine: In Vitro and in Vivo Evaluation. J. Biomat. Sci. Polym. Edit..

[33] Rashid H., Ahmad M., Minhas M.U., Sohail M., Aamir M.F. (2015). Synthesis and Characterization of Poly(hydroxyethyl methacrylate-co-methacrylic acid) Cross Linked Polymeric Network for the Delivery of Analgesic Agent. J. Chem. Soc. Pak..

[34] Liu C., Chen Y., Chen J. (2010). Synthesis and characteristics of pH-sensitive semi-interpenetrating polymer network hydrogels based on konjac glucomannan and poly (aspartic acid) for in vitro drug delivery. Carbohydr. Polym..

[35] Shoaib M.H., Tazeen J., Merchant H.A., Yousuf R.I. (2006). Evaluation of drug release kinetics from ibuprofen matrix tablets using HPMC. Pak. J. Pharm. Sci..

[36] Maziad N.A., El-Hamouly S., Zied E., El-Kelani T.A., Nasef N.R. (2015). Radiation preparation of smart hydrogel has antimicrobial properties for controlled release of ciprofloxacin in drug delivery systems. Asian J. Pharm. Clin. Res..

[37] Samanta H.S., Ray S.K. (2014). Synthesis, characterization, swelling and drug release behavior of semi-interpenetrating network hydrogels of sodium alginate and polyacrylamide. Carbohydr. Polym..

[38] Dergunov S.A., Nam I.K., Mun G.A., Nurkeeva Z.S., Shaikhutdinov E.M. (2005). Radiation synthesis and characterization of stimuli-sensitive chitosan-polyvinyl pyrrolidone hydrogels. Radiat. Phys. Chem..

[39] Estrada-Villegas G., Morselli G., Oliveira M., Gonzalez-Perez G., Lugão A. (2019). PVGA/Alginate-AgNPs hydrogel as absorbent biomaterial and its soil biodegradation behavior. Polym. Bull..

[40] Moharram M.A., Khafagi M.G. (2007). Application of FTIR spectroscopy for structural characterization of ternary poly(acrylic acid)-metal-poly(vinyl pyrrolidone) complexes. J. Appl. Polym. Sci..

[41] Omwoyo W.N., Moloto M.J. (2019). Encapsulation of ibuprofen into solid lipid nanoparticles for controlled and sustained release using emulsification solvent evaporation technique. J. Nanomed. Nanotechnol..

[42] Potta S.G., Minemi S., Nukala R.K., Peinado C., Lamprou D.A., Urquhart A., Douroumis D. (2011). Preparation and characterization of ibuprofen solid lipid nanoparticles with enhanced solubility. J. Microencapsul..

[43] Sarfraz R.M., Khan M.U., Mahmood A., Akram M.R., Minhas M.U., Qaisar M.N., Ali M.R., Ahmad H., Zaman M. (2020). Synthesis of co-polymeric network of carbopol-g-methacrylic acid nanogels drug carrier system for gastro-protective delivery of ketoprofen and its evaluation. Polym. Plast. Tech. Mat..

[44] Shen Y.W., Lin H.T., Gao W.S., Li M.L. (2020). The effects of humic acid urea and polyaspartic acid urea on reducing nitrogen loss compared with urea. J. Sci. Food Agr..

[45] Soares J.P., Santos J.E., Chierice G.O., Cavalheiro E.T.G. (2004). Thermal behavior of alginic acid and its sodium salt. Eclética Química.

[46] Barkat K., Ahmad M., Minhas M.U., Malik M.Z., Sohail M. (2014). Development of a simple chromatographic method for the determination of piracetam in human plasma and its pharmacokinetic evaluation. Drug Res..

[47] Weiss I.M., Muth C., Drumm R., Kirchner H.O.K. (2018). Thermal decomposition of the amino acids glycine, cysteine, aspartic acid, asparagine, glutamic acid, glutamine, arginine and histidine. BMC Biophys..

[48] Sarmento B., Ferreira D., Veiga F., Ribeiro A. (2006). Characterization of insulin-loaded alginate nanoparticles produced by ionotropic pre-gelation through DSC and FTIR studies. Carbohyd. Polym..

[49] Barkat K., Ahmad M., Minhas M.U., Khalid I. (2017). Oxaliplatin-loaded crosslinked polymeric network of chondroitin sulfate-co-poly(methacrylic acid) for colorectal cancer: Its toxicological evaluation. J. Appl. Polym. Sci..

[50] Bianchi S.E., Angeli V.W., de Souza K.C.B., Miron D.D., Carvalho G.D., dos Santos V., Brandalise R.N. (2011). Evaluation of the Solubility of the HPMC/PVA Blends in Biological Fluids in vitro. Mater. Res..

[51] Sadeghi M., Hosseinzadeh H. (2010). Synthesis and super-swelling behavior of a novel low salt-sensitive protein-based superabsorbent hydrogel: Collagen-g-poly(AMPS). Turk. J. Chem..

[52] Suhail M., Wu P.C., Minhas M.U. (2020). Using Carbomer-Based Hydrogels for Control the Release Rate of Diclofenac Sodium: Preparation and In Vitro Evaluation. Pharmaceuticals.

[53] Khan S., Ranjha N.M. (2014). Effect of degree of cross-linking on swelling and on drug release of low viscous chitosan/poly(vinyl alcohol) hydrogels. Polym. Bull..

[54] Suhail M., Khan A., Rosenholm J.M., Minhas M.U., Wu P.C. (2021). Fabrication and Characterization of Diclofenac Sodium Loaded Hydrogels of Sodium Alginate as Sustained Release Carrier. Gels.

[55] Suhail M., Wu P.C., Minhas M.U. (2021). Development and characterization of pH-sensitive chondroitin sulfate-co-poly (acrylic acid) hydrogels for controlled release of diclofenac sodium. J. Saudi Chem. Soc..

[56] Peppas N.A., Sahlin J.J. (1989). A simple equation for the description of solute release. III. Coupling of diffusion and relaxation. Int. J. Pharm..

[57] Singh B., Sharma N. (2009). Mechanistic Implication for Cross-Linking in Sterculia-Based Hydrogels and Their Use in GIT Drug Delivery. Biomacromolecules.

[58] Khan K.U., Minhas M.U., Sohail M., Badshah S.F., Abdullah O., Khan S., Munir A., Suhail M. (2021). Synthesis of PEG-4000-co-poly (AMPS) nanogels by cross-linking polymerization as highly responsive networks for enhancement in meloxicam solubility. Drug Dev. Ind. Pharm..

[59] Mahmood A., Ahmad M., Sarfraz R.M., Minhas M.U. (2018). Development of Acyclovir Loaded -Cyclodextrin-g-Poly Methacrylic Acid Hydrogel Microparticles: An In Vitro Characterization. Adv. Polym. Tech..

[60] Mahmood A., Ahmad M., Sarfraz R.M., Minhas M.U., Yaqoob A. (2016). Formulation and in Vitro Evaluation of Acyclovir Loaded Polymeric Microparticles: A Solubility Enhancement Study. Acta. Pol. Pharm..

[61] Sarfraz R.M., Khan H.U., Mahmood A., Ahmad M., Maheen S., Sher M. (2015). Formulation and evaluation of mouth disintegrating tablets of atenolol and atorvastatin. Indian J. Pharm. Sci..

[62] Nanda S., Sood N., Reddy B.V.K., Markandeywar T.S. (2013). Preparation and Characterization of Poly(vinyl alcohol)-chondroitin Sulphate Hydrogel as Scaffolds for Articular Cartilage Regeneration. Indian J. Mater. Sci..

